# Fast and low-cost evaluation of hydroxykynurenine activity

**DOI:** 10.1016/j.mex.2020.100982

**Published:** 2020-07-02

**Authors:** Larissa G. Maciel, Janaína V. dos Anjos, Thereza A. Soares

**Affiliations:** Department of Fundamental Chemistry, Federal University of Pernambuco (UFPE), Brazil

**Keywords:** Kynurenine pathway, HKT activity, Inhibition assay

## Abstract

The enzyme 3-hydroxykynurenine transaminase (HKT) acts as an important enzyme in tryptophan catabolism of disease-carrier insects, e.g. *Aedes aegypti* and *Anopheles gambiae*. HKT is a detoxification enzyme that converts 3-hydroxykynurenine (a precursor for reactive nitrogen and oxygen species) into xanthurenic acid (stable and nontoxic compound). We have previously synthesized eleven new oxadiazole derivatives and demonstrated their noncompetitive inhibitory activity towards HKT from *A. aegypti* (https://doi.org/10.1016/j.bmc.2019.115252). These findings are presented in a research paper accompanying the present technical report on a new assay to overcome the fact that the substrate and product of the HKT-catalyzed reaction exhibit maximum absorption at very near wavelength (370 and 369 nm, respectively). The methods previously described in the literature rely on chromatographic separation prior to absorbance quantification, which limits their use for inhibitor screening. Due to HKT attractive features as a molecular target for larvicidal compounds, we report herein a new, faster and affordable methodology to evaluate the enzymatic activity of recombinant HKT, and therefore allow for the fast screening of potential HKT inhibitors via absorbance spectrophotometer. The advantages of the proposed methodology to previously described ones are:

• It is faster and cheaper than HPLC-based assays because it does not require the use of chromatography columns and solvents to separate reaction components;
• It uses of 96-well plates, enabling the simultaneous quantification of samples;
• It can be applied to all transaminases that have xanthurenic acid as a product.

## Graphical abstract

**Figure:**
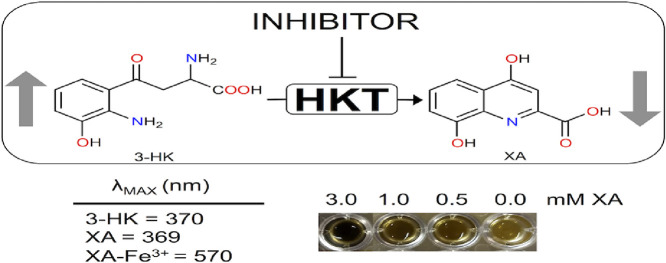

Specifications table

**Table:** 

Subject Area:	*Chemistry*
More specific subject area:	*Enzyme activity and inhibition assay.*
Method name:	*Detection of xanthurenic acid – Fe^3+^ complex in enzymatic assay.*
Name and reference of original method:	Evaluation of HKT activity from *Aedes aegypti* and *Anopheles gambiae* via HPLC-UV and LC-MS/MS methods
	[3] Q. Han, J. Li, Comparative characterization of *Aedes* 3-hydroxykynurenine transaminase/alanine glyoxylate transaminase and *Drosophila* serine pyruvate aminotransferase, FEBS Lett. 527 (2002) 199–204. https://doi.org/10.1016/S0014–5793(02)03,229–5.
	[8] R. Canavesi, R. Miggiano, M. Stella, U. Galli, F. Rossi, M. Rizzi, E. Del Grosso, Study of *Anopheles gambiae* 3-hydroxykynurenine transaminase activity and inhibition by LC-MS/MS method, J. Pharm. Biomed. Anal. 173 (2019) 154–161. https://doi.org/10.1016/j.jpba.2019.05.025.
Resource availability:	*All reagents were purchased in Sigma-Aldrich, except: 1* *M HEPES* pH *7.5 – Gibco©, Iron (III) chloride hexahydrate – Dinâmica^Ⓡ^ and Recombinant HKT – Expressed in house.*
	*Set of adjustable-volume micropipettes and tips.*
	*Microcentrifuge and conical tubes for reagent dilutions.*
	*Nunc™ 96-Well Polystyrene Round Bottom Microwell Plates from Thermo Scientific™*
	*ELx808 BIOTEK© or any absorbance spectrophotometer with measurements at 570 nm*
	*GraphPad Prism 7.0 or any graphical software*

## Introduction

The search for new chemical insecticides has gained importance in recent years due to the growing outbreaks of arboviruses (Yellow Fever, Dengue, Zika and Chikungunya Fever) and the regular frequency of the occurrence of malaria in tropical regions [1]. Most insects have exhibited some kind of resistance to known insecticides [2], stimulating the research for new biological targets and, consequently, new classes of insecticides to be developed. A particular biochemical route in these arboviruses vectors has been shown to be a potential molecular target, the so-called kynurenine pathway, which is the main route for metabolizing tryptophan in insects [3,4]. In this pathway, tryptophan is oxidized to quinolinic or kynurenic acid or xanthurenic acid (XA) depending on the organism. In mosquitoes, the chemically stable XA is biosynthesized from 3-hydroxykynurenine in a process catalyzed by 3-hydroxykynurenine transaminase (HKT), the sole enzyme responsible for 3-HK regulation in mosquitoes [5], [6], [7], [8]. Therefore, the inhibition of the enzymatic conversion of 3-HK into xanthurenic acid provides in a powerful approach to control *A. aegypti* development via the accumulation of 3-HK, a toxic metabolite to living organisms, since it can be spontaneously oxidized into reactive oxygen species, leading to oxidative stress in mosquito's neuronal cells [4].

We have previously identified the enzyme HKT as a potential molecular target for inhibition by oxadiazole derivatives [6]. This finding has encouraged us to develop a reliable method capable to measure HKT inhibition without separation of 3-HK and XA, which have maximum absorption at very near wavelength (*λ*_max_ = 370 and 369 nm, respectively). This new approach offers a rapid, low-cost and more straightforward means to screen for HKT antagonists. In what follows, we describe the standardization of an absorbance-based biochemical protocol to evaluate HKT activity by means of XA-Fe^3+^ complex (*λ*_max_ = 570 nm). The assays were performed on a 96-well round bottom microplate, allowing simultaneous reading of multiple samples in a characterization of HKT activity. The formed xanthurenic acid is detected as a complex with Fe^+3^, first described by Lepkovski and coworkers to detect XA in pyridoxine-deficient mice [9]. This colorimetric test abolishes the use of organic solvents as mobile phase, equipment and chromatography columns, thus making the assay less expensive than LC-based methods established in earlier literature [3,8]. The analysis of HKT activity is faster in colorimetric assays because there is no requirement to separate reaction components prior to detection of xanthurenic acid, removing the runtime of total time of analysis. The colorimetric test described herein is also almost universal, once different types of inhibitors can be tested with exception to highly-conjugated organic compounds that absorbs in visible region of ultraviolet spectra [10].

## Method details

All solutions were freshly prepared except for 25 mM 3-D,L-hydroxykynurenine solution, which was prepared previously and then stocked at −20 °C.

Stock solutions:

• 0.1 M HCl
• 0.1 M NaOH
• HEPES Buffer:

10 mL of 1 M HEPES-NaOH pH 7.5
1.25 mL of 4 M NaCl
Ultrapure H_2_O up to 50 mL

Stock reagents:

• 100 mM Iron (III) chloride hexahydrate: 135.2 mg in 0.1 M HCl up to 5 mL
• 20 mM Pyridoxal-5′-phosphate hydrate: 34.3 mg in 0.1 M NaOH up to 7 mL

Working reagents:

• 25 mM 3-D,L-hydroxykynurenine (3-HK) solution: 11.22 mg in HEPES Buffer up to 2 mL
• 20 mM Xanthurenic acid (XA) solution: 16.4 mg in 0.1 M NaOH up to 4 mL
• 0.5 mM Pyridoxal-5′-phosphate hydrate (PLP) solution: 0.25 mL of 20 mM stock reagent in HEPES Buffer up to 10 mL
• 50 mM Sodium Pyruvate solution: 55 mg in HEPES Buffer up to 10 mL
• 10 mM Iron (III) chloride hexahydrate (Fe^3+^) solution: 1 mL of 100 mM stock reagent in 0.1 M HCl up to 10 mL
• 0.1 µg/µL recombinant 3-hydroxykynurenine transaminase (HKT) from *Aedes aegypti*.

## HKT activity measurement

The experiments were performed in a round-bottom 96-well transparent plate and the samples have a final volume of 100 µL. Each reaction contains 2 µg of HKT, 2 mM 3-HK solution, 2 mM Sodium pyruvate solution, 40 µM PLP solution and HEPES Buffer to complete the reaction volume. Mixes were designed to ensure that the reaction only starts when the enzyme is added into the reaction. For this reason, Mix 1 contains the enzyme and its cofactor PLP and Mix 2, the others reaction components. Mix 3 contains only PLP and HEPES Buffer and it is used to compose the blank. Therefore, the blank is made by the combination of Mix 3 (28 µL) and Mix 2 (72 µL) and the sample is made by the combination of Mix 1 (28 µL) and Mix 2 (72 µL), and each experiment has a total volume of 100 µL. Each sample or blank should be analyzed, at least, in duplicate.

**Table:** 

Mix 1:			
**Reagent**	**Initial concentration**	**Volume (µL)**	**Final concentration**
HKT	0.1 µg/µL	20	2 µg
PLP	500 µM	8	40 µM
	**Total:**	28 µL	
Mix 2:			
**Reagent**	**Initial concentration**	**Volume (µL)**	**Final concentration**
3-HK	25 mM	8	2 mM
Pyruvate	50 mM	4	2 mM
Buffer	–	60	–
	**Total:**	72 µL	
Mix 3:			
**Reagent**	**Initial concentration**	**Volume (µL)**	**Final concentration**
Buffer	–	20	–
PLP	500 µM	8	40 µM
	**Total:**	28 µL	

In order to determine the total reaction time, it is necessary to characterize the progress curve. This curve will give information about the reaction kinetics. In our experiments, it can be visualized three regions: a linear part that shows a first-order dependence between concentration and time (0–5 min); a transition region (5–15 min); and a plateau region (15–25 min), that is reached when chemical equilibrium takes place. Therefore, the assay must be performed in a time interval to allow the differentiation of these three distinct regions, once the region of interest *de facto* is only the first one (Fig. 2A).

To decrease error between replicates is recommended calculating the total number of samples required to the experiment and then preparing each mix to contain “*n* + 1” samples to ensure that there will be enough reagents for all experiments to be performed.

E.g.: Comparison of the activity between 3 batches of purified HKT in duplicate. To calculate the rate, it is necessary to perform reads in two periods of time (in this case, 0 and 5 min) (Table 1).
Mix 1: 3 batches x 2 replicates x 2 periods of time = 12 samples
Mix 2: 3 batches x 2 replicates x (2 periods of time + 1 blank) = 18 samples
Mix 3: 3 batches x 2 replicates x 1 blank = 6 samples

Therefore, the total volume of Mix 1 must be multiplied by 13, the total volume of Mix 2 by 19 and the total volume of Mix 3 by 7.

After preparing all the mixes, the following steps have to be executed:

1. Set the heater at 50 °C.
2. Add 72 µL of Mix 2 in each well.
3. Add 28 µL of Mix 3 in BLANK and 28 µL of Mix 1 in SAMPLES.
4. Add 100 µL of 10 mM Fe^3+^ in BLANK and SAMPLES of TIME 0 min.
5. Read the plate at 570 nm.
6. Incubate the plate at 50 °C for 5 min.
7. Add 100 µL of 10 mM Fe^3+^ in SAMPLES of TIME 5 min.
8. Read the plate at 570 nm.

## Calibration curve

**Fig. 1:**
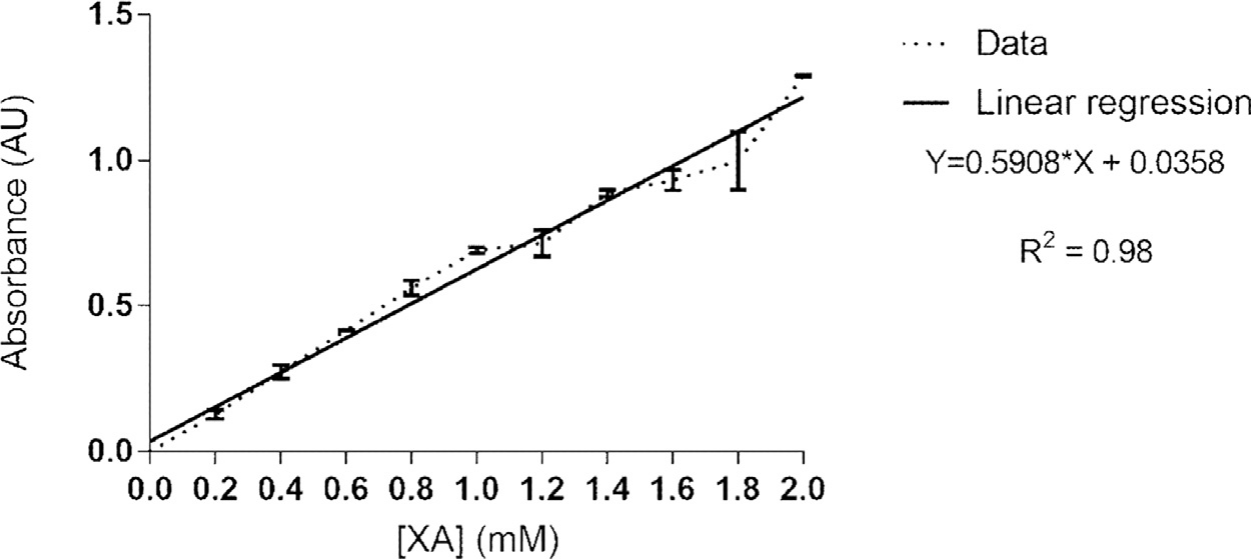
Calibration curve used for quantification of biochemical and inhibition data. Each sample contains 2 mM sodium pyruvate, 40 µM PLP, 10 mM FeCl_3_·6H_2_O in 0.1 M HCl and variable concentration of XA (0–2 mM) in a final volume of 200 µL.

A calibration curve must be executed to quantify the amount of xanthurenic acid generated by HKT. The samples contain both mix A (with fixed Pyruvate and PLP concentrations) and mix B (with variable XA concentration) in a final volume of 100 µL. Each XA concentration must be read in triplicate. A linear regression of the data generates the calibration curve (Fig. 1).

Mix A (for all wells):

**Table:** 

**Reagent**	**Initial concentration**	**Volume (µL)**	**Final concentration**
Pyruvate	50 mM	4	2 mM
PLP	500 µM	8	40 µM
	**Total:**	12 µL	

Mix B (for each XA concentration):

**Table:** 

**Final concentration XA (mM)**	**Volume XA (µL)**	**Volume Buffer (µL)**
0.0	–	88
0.2	1	87
0.4	2	86
0.6	3	85
0.8	4	84
1.0	5	83
1.2	6	82
1.4	7	81
1.6	8	80
1.8	9	79
2.0	10	78
4.0	20	68
6.0	30	58
8.0	40	48
**Total in each well:**	88 µL	

After preparing all the mixes, the following steps have to be executed:

1. Add 12 µL of Mix A in each well.
2. Add 88 µL of Mix B in the respective wells.
3. Add 100 µL of 10 mM Fe^3+^ in all samples.
4. Read the plate at 570 nm.

## Enzyme kinetics

**Fig. 2:**
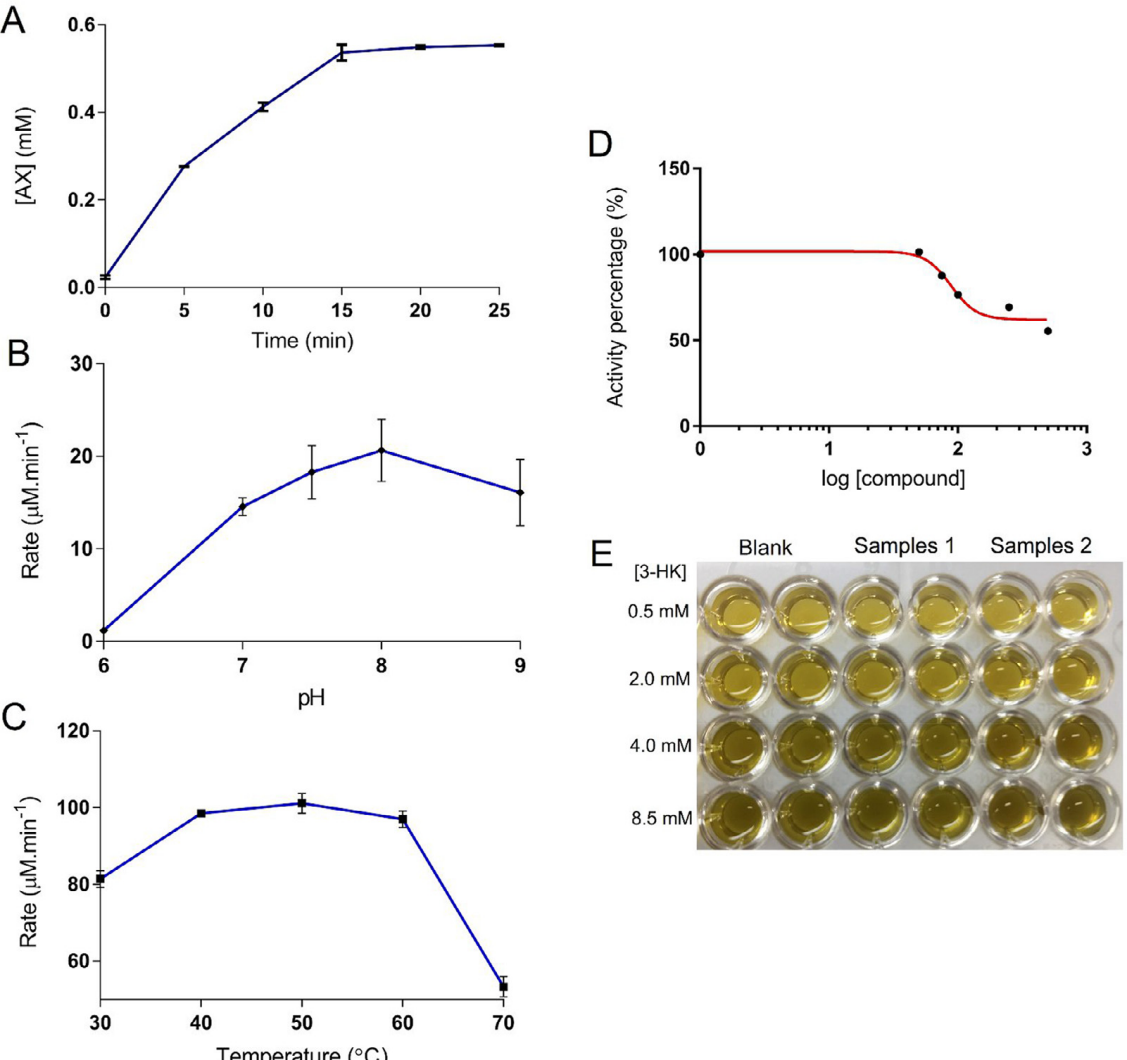
The progress curve (A), pH evaluation in buffers Tris pH 6,7 and 8, HEPES pH 7.5 and Borate pH 9 (B), temperature evaluation (C), the non-linear regression curve of inhibition data from the compound sodium 4-[3-(*p*-*tolyl*)−1,2,4-*oxadiazol*-5-*yl*]butanoate to estimate its IC_50_(D) and a picture of the plate after a experiment (E).

The estimation of the kinetics constants can be performed by adjusting the amount (or volume) of 3-HK, Pyruvate and Buffer solutions in Mix 2. In these experiments, we tested four different 3-HK concentrations (0.5, 2.0, 4.0 and 8.5 mM) to determine the kinetics constants [5] (Fig. 2E). The preparation of the mixes and general procedures are similar to described in “HKT activity measurements” section.

## pH evaluation

The pH screening can be executed by replacing the Buffer described in “Stock solutions” subsection by any buffer of your choice. In our experiments, the assay was tested in pH ranging from 6 to 9 in different buffers (Tris–HCl, HEPES and Borate Buffers), but the enzyme has its best activity between pH 7 and 9. As HKT theoretical isoelectric point is around 6, possibly this is the reason there is no relevant activity at this point (Fig. 2B). The preparation of the mixes and general procedures are the same as described in “HKT activity measurements” section.

## Temperature evaluation

The temperature screening can also be evaluated by changing the incubation temperature. This methodology was already tested in temperatures from 30 to 70 °C, yielding comparable results with the literature (Fig. 2C). The preparation of the mixes and general procedures are the same as described in “HKT activity measurements” section.

## Inhibition assays

In order to test compounds as HKT inhibitors, the preparation of the mixes and general procedures are slightly different from the section “HKT activity measurements”. The methodology requires a pre-incubation of Mix 1 and Mix 3 with the testing compound at 50 °C for 30 min. Then, Mix 2 is added and the experiment may continue. Mixes 1 and 3 have to be prepared for each concentration of the tested compound. Each sample or blank must be read at least in duplicate. Blank and negative control (assay without any testing compound) must be executed in the same lineup as the samples. To estimate IC_50_ of each compound is recommended testing at least 5 concentrations (Fig. 2D). The suggestion of setting the mixes to “*n* + 1” samples is also valid in this section.

Mix 1:

**Table:** 

**Reagent**	**Initial concentration**	**Volume (µL)**	**Final concentration**
HKT	0.1 µg/µL	20	2 µg
PLP	500 µM	8	40 µM
Compound	*	30	*
	**Total:**	58 µL	

Mix 2:

**Table:** 

**Reagent**	**Initial concentration**	**Volume (µL)**	**Final concentration**
3-HK	25 mM	8	2 mM
Pyruvate	50 mM	4	2 mM
Buffer	–	30	–
	**Total:**	42 µL	

Mix 3:

**Table:** 

**Reagent**	**Initial concentration**	**Volume (µL)**	**Final concentration**
Buffer	–	20	–
PLP	500 µM	8	40 µM
Compound	*	30	*
	**Total:**	58 µL	

*The initial concentration of the testing compound must be **3.33 times** higher than the desired final concentration (adjusted into 30 µL of 100 µL). This is the ratio between the initial and final concentration to reach the standardized volumes (30 µL of testing compound in a total of 100 µL of reaction).

After preparing mixes 1 and 3, the following steps have to be executed:

1. Set the water bath at 50 °C.
2. Incubate mixes 1 and 3 at 50 °C for 30 min.
3. In meanwhile, prepare Mix 2.
4. Set the heater at 50 °C.
5. Add 42 µL of Mix 2 in each well
6. Add 58 µL of Mix 3 in BLANK and 58 µL of Mix 1 in SAMPLES.
7. Add 100 µL of 10 mM Fe^3+^ in BLANK and SAMPLES of TIME 0 min.
8. Read the plate at 570 nm.
9. Incubate the plate at 50 °C for 5 min.
10. Add 100 µL of 10 mM Fe^3+^ in SAMPLES of TIME 5 min.
1.1 Read the plate at 570 nm.

## Method validation

The characterization of recombinant HKT from *Aedes aegypti* expressed in *Escherichia coli* was achieved using this methodology. The results are comparable to those related in the literature for the same enzyme expressed in insect cells. Several experiments performed with HKT in this assay conditions are displayed as examples (Fig. 2).

## Conclusion

We have described a new, fast and affordable methodology to evaluate recombinant HKT activity which allows for the screening potential HKT inhibitors via absorbance spectrophotometer. This method is based on the complexation reaction between xanthurenic acid (product of conversion of 3-kydroxykynurenine by HKT) with ferric ion in a 96-well round bottom microplate, allowing simultaneous reading of multiple samples. Attention must be paid to the solutions stock conditions particularly to the 3-HK solution, since this is an unstable molecule and its oxidation products could interfere in the formation of XA-Fe^3+^ complex or HKT catalysis [11]. To overcome this issue, these solutions must be freshly prepared and stocked at low temperatures even during the experiments. Another drawback could be the pH evaluation above 9, as mentioned in our previous work [5]. The conditions described here prevent the formation of insoluble Fe^3+^ species which lead to anomalous absorbances and/or false positives. However, these extreme values of pHs are not the common optimal pH for HKT enzymes [5,7]. To the best of our knowledge, this is the fastest and cheapest method of screening for new HKT inhibitors ever reported, with an actual cost per sample 7 times smaller than the previous HPLC-based methodology.

**Table 1:** Visual example of a plate in comparative rate experiment between 3 batches of purified HKT performed in duplicate.

	Blank 1	Blank 2	0 min - 1	0 min - 2	5 min - 1	5 min - 2
**Batch 1**	Mix 3 + 2	Mix 3 + 2	Mix 1 + 2	Mix 1 + 2	Mix 1 + 2	Mix 1 + 2
**Batch 2**	Mix 3 + 2	Mix 3 + 2	Mix 1 + 2	Mix 1 + 2	Mix 1 + 2	Mix 1 + 2
**Batch 3**	Mix 3 + 2	Mix 3 + 2	Mix 1 + 2	Mix 1 + 2	Mix 1 + 2	Mix 1 + 2

## Declaration of Competing Interests

The authors declare that they have no known competing financial interests or personal relationships that could have appeared to influence the work reported in this paper.
